# Geometric and dosimetric impact of 3D generative adversarial network-based metal artifact reduction algorithm on VMAT and IMPT for the head and neck region

**DOI:** 10.1186/s13014-021-01827-0

**Published:** 2021-06-06

**Authors:** Mitsuhiro Nakamura, Megumi Nakao, Keiho Imanishi, Hideaki Hirashima, Yusuke Tsuruta

**Affiliations:** 1 Division of Medical Physics, Department of Information Technology and Medical Engineering, Human Health Sciences, Graduate School of Medicine, Kyoto University, 53 Kawahara-cho, Shogoin, Sakyo-ku, Kyoto, 606-8507 Japan; 2 Department of Radiation Oncology and Image-Applied Therapy, Graduate School of Medicine, Kyoto University, Kyoto, Japan; 3 Department of Systems Science, Graduate School of Informatics, Kyoto University, Kyoto, Japan; 4 e-Growth Co., Ltd., Hyogo, Japan; 5 Division of Clinical Radiology Service, Kyoto University Hospital, Kyoto, Japan

**Keywords:** 3D GAN, Metal artifact reduction, VMAT and IMPT, Head and neck cancer

## Abstract

**Background:**

We investigated the geometric and dosimetric impact of three-dimensional (3D) generative adversarial network (GAN)-based metal artifact reduction (MAR) algorithms on volumetric-modulated arc therapy (VMAT) and intensity-modulated proton therapy (IMPT) for the head and neck region, based on artifact-free computed tomography (CT) volumes with dental fillings.

**Methods:**

Thirteen metal-free CT volumes of the head and neck regions were obtained from The Cancer Imaging Archive. To simulate metal artifacts on CT volumes, we defined 3D regions of the teeth for pseudo-dental fillings from the metal-free CT volumes. HU values of 4000 HU were assigned to the selected teeth region of interest. Two different CT volumes, one with four (m4) and the other with eight (m8) pseudo-dental fillings, were generated for each case. These CT volumes were used as the *Reference*. CT volumes with metal artifacts were then generated from the Reference CT volumes (*Artifacts*). On the Artifacts CT volumes, metal artifacts were manually corrected for using the water density override method with a value of 1.0 g/cm^3^ (*Water*). By contrast, the CT volumes with reduced metal artifacts using 3D GAN model extension of CycleGAN were also generated (*GAN-MAR*). The structural similarity (SSIM) index within the planning target volume was calculated as quantitative error metric between the Reference CT volumes and the other volumes. After creating VMAT and IMPT plans on the Reference CT volumes, the reference plans were recalculated for the remaining CT volumes.

**Results:**

The time required to generate a single GAN-MAR CT volume was approximately 30 s. The median SSIMs were lower in the m8 group than those in the m4 group, and ANOVA showed a significant difference in the SSIM for the m8 group (*p* < 0.05). Although the median differences in D_98%_, D_50%_ and D_2%_ were larger in the m8 group than the m4 group, those from the reference plans were within 3% for VMAT and 1% for IMPT.

**Conclusions:**

The GAN-MAR CT volumes generated in a short time were closer to the Reference CT volumes than the Water and Artifacts CT volumes. The observed dosimetric differences compared to the reference plan were clinically acceptable.

## Background

Computed tomography (CT) systems are widely used in clinical practice. Artifacts such as motion, ring and metal artifacts [1] are commonly encountered in clinical CT and may reduce the image quality. Metal artifacts appear as streaking artifacts and dark bands in the reconstructed images owing to photon starvation and beam-hardening effects under the presence of metals in the subject. The shape of the metal and its surroundings are blurred in metal artifacts, leading to inaccurate Hounsfield unit (HU) values and the poor visualization of internal organs [2]. These drawbacks reduce treatment accuracy in radiotherapy, consequently leading to radiation-induced toxicities [3].

Several sinogram-based metal artifact reduction (MAR) algorithms, such as orthopaedic MAR (O-MAR; Philips Healthcare System, Cleveland, OH, USA) [4], iterative MAR (iMAR; Siemens Healthcare, Forchheim, Germany) [5], single-energy MAR (SEMAR; Canon Medical Systems, Otawara, Japan) [6], SmartMAR (GE Medical Systems, Waukesha, WI, USA) [7], and virtual monochromatic images via dual-energy CT [8], are clinically available. Although insufficient correction of sinograms around metals lead to additional artifacts and image blurring, these MAR algorithms generally yield clinically acceptable CT images in terms of image quality and HU value accuracy [9–13]. However, a notable shortcoming is that it is impossible to examine the true effect of MAR algorithms due to lack of real patients’ data without metal artifacts.

With recent developments in artificial intelligence, deep learning (DL) has attracted attention in the field of medicine. DL has been mainly employed in image-based MAR algorithms [14]. Most DL-MAR algorithms are supervised methods that require paired data. However, a significant drawback of supervised methods is difficulty in collecting them from real patients. To overcome this issue, generative adversarial networks (GANs) have been extensively studied as a framework for unsupervised methods [15]. Recently, GAN-based MAR algorithms for images with metals has attracted significant attention in diagnostic radiology [16]. In therapeutic radiology, Koike et al. have proposed a two-dimensional (2D) cycle-consistent GAN (CycleGAN)-based MAR method in intensity-modulated radiotherapy for head and neck cancer patients with dental fillings [17]. They demonstrated the efficiency of the planning process by eliminating manual delineation and consistent dose distribution against metal artifacts. However, their evaluation was based on 2D analyses using artifact-corrected CT volumes with the water density override method. Although 3D analyses were performed in a recent phantom study by Branco et al. [13], the effects of MAR are unclear in real-patient datasets, as artifact-free CT volumes with dental fillings were not available in their study.

We successfully generated CT volumes with metal artifacts from artifact-free CT volumes with dental fillings. In addition, we reduced artifacts by employing a 3D GAN-based MAR, which is an extension of the image-to-image translation framework of CycleGAN [18]. Thus, artifact-free CT volumes with dental fillings can be treated as ground truths. In this study, we investigated the geometric and dosimetric impacts of the 3D GAN-based MAR algorithm on volumetric-modulated arc therapy (VMAT) and intensity-modulated proton therapy (IMPT) for the head and neck regions, based on the artifact-free CT volumes with dental fillings.

## Methods

### CT volumes

Thirteen metal-free CT volumes of the head and neck regions were obtained from The Cancer Imaging Archive [19]. The field of view (350–400 mm), matrix size (512 × 512), and slice thickness (1–3 mm) were clinically acceptable for treatment planning CT.

**Fig. 1:**
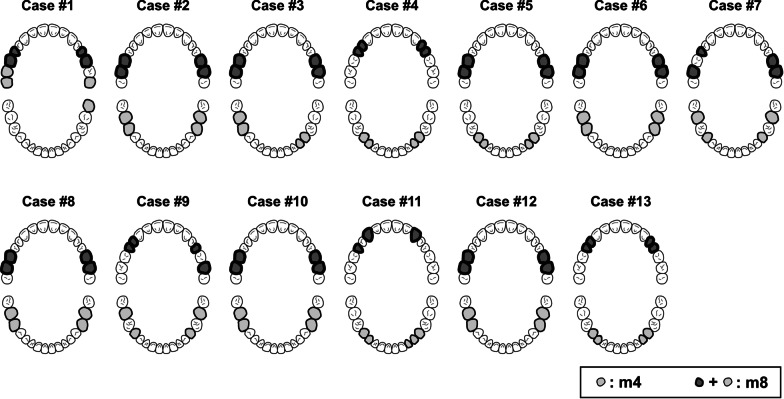
Schemas of dental arch for each case. Teeth painted by light gray are dental fillings in the m4 group, and by both light and dark gray are in m8 groups

To simulate metal artifacts on CT volumes, we defined 3D regions of the teeth for pseudo-dental fillings from the metal-free CT volumes. Figure 1 illustrates the scheme of dental arches, showing the pseudo-dental fillings for each case. HU values of 4000 HU were uniformly assigned to the selected teeth region of interest (ROI). Two different CT volumes, one with four (m4) and the other with eight (m8) pseudo-dental fillings, were generated for each case. These CT volumes were used as the *Reference* in this study.

### Generation of metal artifacts and manual correction of metal artifacts

The metal artifacts were simulated based on the procedure and parameters used in [14], as follows: (1) simulated sinograms were obtained from the Reference CT volumes by forward projection, and (2) the CT volumes were reconstructed using filtered back projection from the simulated sinograms. The number of projection views over a rotation and detector bins were 984 and 920, respectively. The distance between the X-ray tube source and the rotation center was 59.5 cm. The code for forwarding projection and image reconstruction are publicly available [14]. The resultant CT volumes were labeled *Artifacts.* Metal artifacts were more prominent in the Artifacts CT volumes in the m8 group than in the m4 group.

Metal artifacts were manually corrected on the Artifacts CT volumes by a senior medical physicist using the water density override method with a value of 1.0 g/cm^3^. The m4 and m8 groups were corrected using standard clinical methods. The CT volumes with manually corrected metal artifacts were termed *Water*.

### GAN-based metal artifact reduction

We used a 3D GAN model extension of CycleGAN in this study, the details of which are described in a prior work [18]. Our GAN-MAR approach is based on partial volume-to-partial volume translation and includes two mapping functions—image domain with artifacts to image domain without artifacts (*G*_*Y*_) and vice versa (*G*_*X*_). Two adversarial discriminators *D*_*X*_ and *D*_*Y*_ are also introduced, where *D*_*X*_ aims to distinguish between volumes *x* and *G*_*Y*_(*y*), and *D*_*Y*_ to distinguish *y* from *G*_*X*_(*x*). Here, a training sample *x* or *y* is an unpaired partial volume that consists of *N* spatially continuous image slices.

To generate the CT volumes for artifact correction using 3D GAN (*GAN-MAR*), unpaired data including 300 CT volumes with metal artifacts and 53 CT volumes without metal artifacts were prepared. No labels were assigned to the regions with metal artifacts. Then, CT volumes without metal artifacts were augmented sixfold by 3D rotation and deformation. Nine sequential slices were input to the network, i.e., detection and correction of metal artifacts was conducted based on partial volume-to-partial volume translation. The key parameters at training phase were as follows: the input and output size was 512 × 512 × 9, batch size was 8, and the number of epochs was 1000. Google Compute Engine (CPU: Intel Xeon, Memory: 16 GB, GPU: Tesla T4, Memory: 16 GB) was used for the calculations.

### Treatment planning

**Fig. 2:**
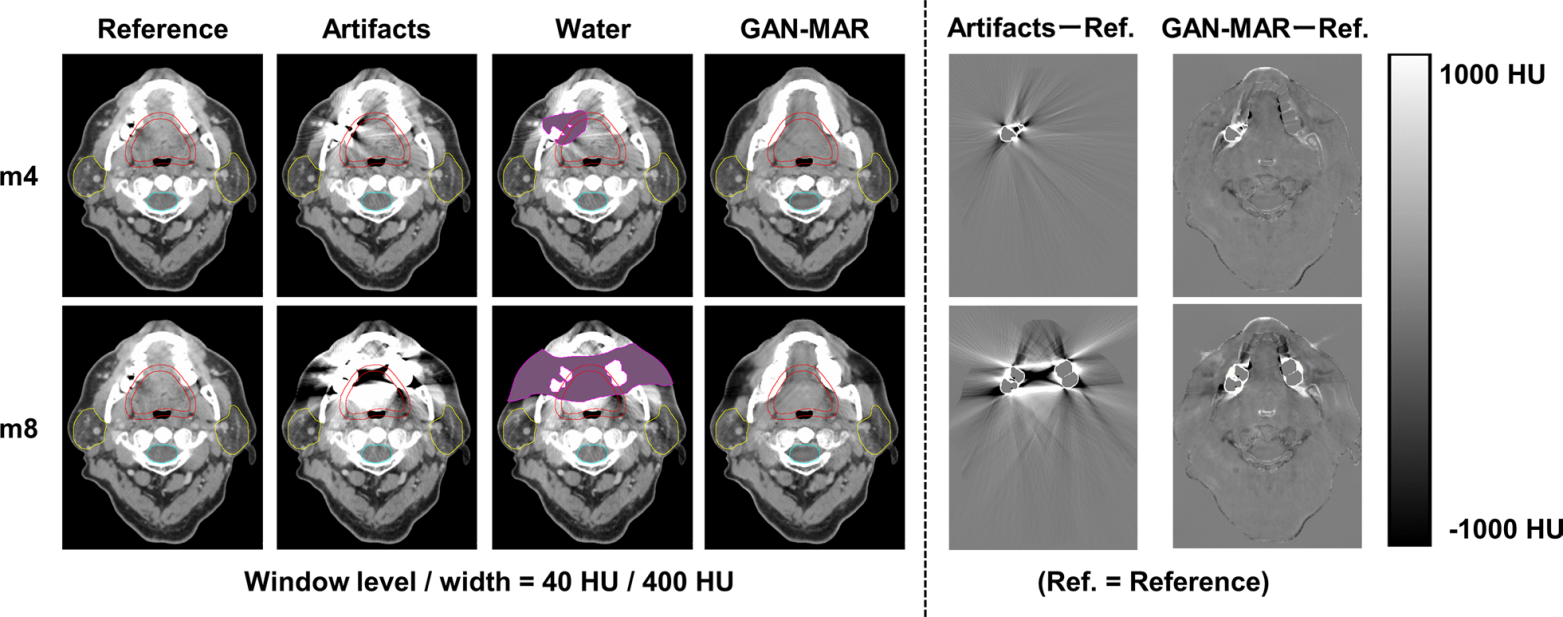
Representative axial slices from Reference, Artifacts, Water, and GAN-MAR CT volumes in (upper) m4 and (lower) m8 groups. Difference images to Reference are also shown

Metal artifacts affect the accuracy of radiotherapy for oropharyngeal cancer. In this study, the pseudo-clinical target volume (CTV) including the base of the tongue was manually delineated on the Reference CT volumes. The planning target volume (PTV) was defined by adding a 5 mm margin to the CTV. The spinal cord and the parotids were also delineated as organs at risk. Figure 2 shows the representative axial and sagittal slices.

One VMAT plan and one IMPT plan were generated on the Reference CT volumes on the Eclipse treatment planning system (ver. 15.6, Varian Medical Systems, Palo Alto, CA, USA) with a grid size of 2.5 mm. The VMAT plan comprised two coplanar arcs of 6 MV photon beams using TrueBeam (Varian Medical Systems). Beam avoidance sectors from 330° to 30° (central angle of 60°) were used to minimize the effect of metal and artifacts on dose distribution. The collimator angles were 330° for arc 1 and 30° for arc 2. The dose distributions were calculated using Acuros XB (Varian). By contrast, spot scanning technique with two incident proton beams of kinetic energies between 70 and 250 MeV delivered by the Varian proton therapy system was used in the IMPT plan. The gantry angles were set to 150° and 210° to divert incident proton beams from the metal. A proximal and distal margin of 5 mm and a lateral margin of 10 mm were added to the PTV, and CTV-based robust optimization was not employed. The Proton Convolution Superposition algorithm (Varian) was used for dose calculation.

**Fig. 3:**
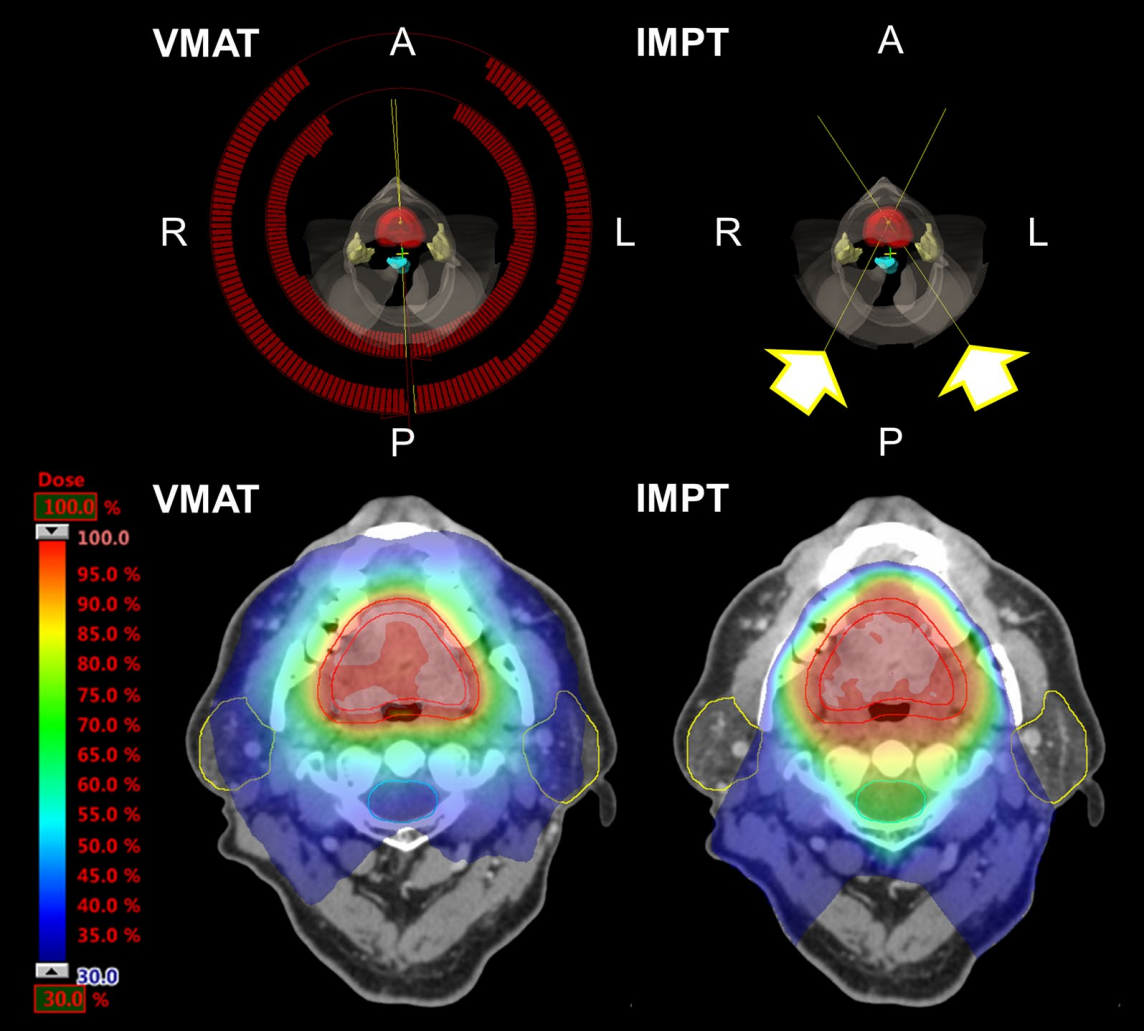
Representative (upper) treatment plan and (lower) dose distribution for (left) VMAT and (right) IMPT plan

The prescribed dose of 60 Gy in 30 fractions was administered to 50% of the PTV (PTV D_50%_) for each plan. The reason for only PTV D_50%_ prescription was that D_50%_ is typically a suitable choice for a representative absorbed-dose value for the PTV [20]. In addition, multiple use of prescriptions made evaluation difficult. A dose covering of 2% of the PTV volume (PTV D_2%_) was set to < 105%, the maximum dose (D_max_) to the spinal cord was < 45 Gy, and the mean dose (D_mean_) to the parotids was < 26 Gy. Figure 3 shows a representative treatment plan and dose distribution for the VMAT and IMPT plan.

### Evaluation

The ROIs of body, CTV, PTV, the spinal cord and the parotids on the Reference CT volumes were rigidly propagated to the Artifacts, Water and GAN-MAR CT volumes in each case (Fig. 2). As these CT volumes shared the origin of the orthogonal coordinate system, the shape and position of the ROIs were identical for these CT volumes. Then, the root mean square error (RMSE) of the HU values and the structural similarity (SSIM) index [21] within the PTV were calculated as quantitative error metrics between the Reference CT volumes and the other volumes, to examine the correlation between the differences in the dose-volumetric indices (DVIs) for the PTV and the RMSE and SSIM within the PTV in the VMAT and IMPT plans. SSIM is in the range of 0 and 1, and a value of 1 indicates perfect structural similarity. One-way analysis of variance (ANOVA) was performed to evaluate significant differences in the RMSE and SSIM. In addition, the reference plans were recalculated on the Water and GAN-MAR CT volumes while maintaining the monitor units and beam arrangements. As the Artifacts CT volumes are not typically used in clinical practice, we did not calculate the associated dose distribution. The difference in the DVIs from the reference plans were calculated. A Mann–Whitney U test was performed to evaluate the statistical significance of the difference in DVIs from the reference plans. During both statistical analyses, the level of significance was set to 0.05, and all statistical analyses were performed using the R (version 3.6.1) package.

## Results

The calculation time required to generate a single GAN-MAR CT volume was approximately 30 s.

The median PTV volume was 133.6 ml (range 98.7–169.1 ml), and median overlap ratio of the artifact corrected volume to the PTV volume was 7.5% (range 0.0–18.5%) in the m4 group and 14.3% (range 0.0–22.8%) in the m8 group.

Table 1 summarizes the RMSE and the SSIM within the PTV. The maximum RMSE was 10.64 HU observed in the m8 group, and reduced in order for the Artifacts, GAN-MAR, and Water CT volumes in both the m4 and m8 groups. There was a significant difference to population means in the RMSE in the m8 group (*p* < 0.05). As can be seen from the SSIM values, the GAN-MAR CT volumes were closer to the Reference CT volumes than the Water and Artifacts CT volumes. The median SSIMs were lower in the m8 group than those in the m4 group, and ANOVA showed a significant difference to population means in the SSIM for the m8 group (*p* < 0.05).

**Table 1:** Summary of RMSE of the HU values and SSIM within the PTV

RMSE (HU)				SSIM			
Artifacts	Water	GAN-MAR	*p* value	Artifacts	Water	GAN-MAR	*p* value
*m4*							
6.87 (0.84–8.45)	4.22 (0.84–6.66)	4.84 (1.33–6.04)	0.13	0.84 (0.75–0.96)	0.84 (0.76–0.96)	0.91 (0.80–0.96)	0.09
*m8*							
8.69 (1.06–10.64)	5.95 (1.05–8.46)	6.24 (1.65–7.34)	< 0.05	0.74 (0.66–0.95)	0.73 (0.65–0.95)	0.86 (0.73–0.92)	< 0.05

Data are shown in median (minimum–maximum)

*RMSE* root mean square error, *SSIM* structural similarity, *PTV* planning target volume

**Fig. 4:**
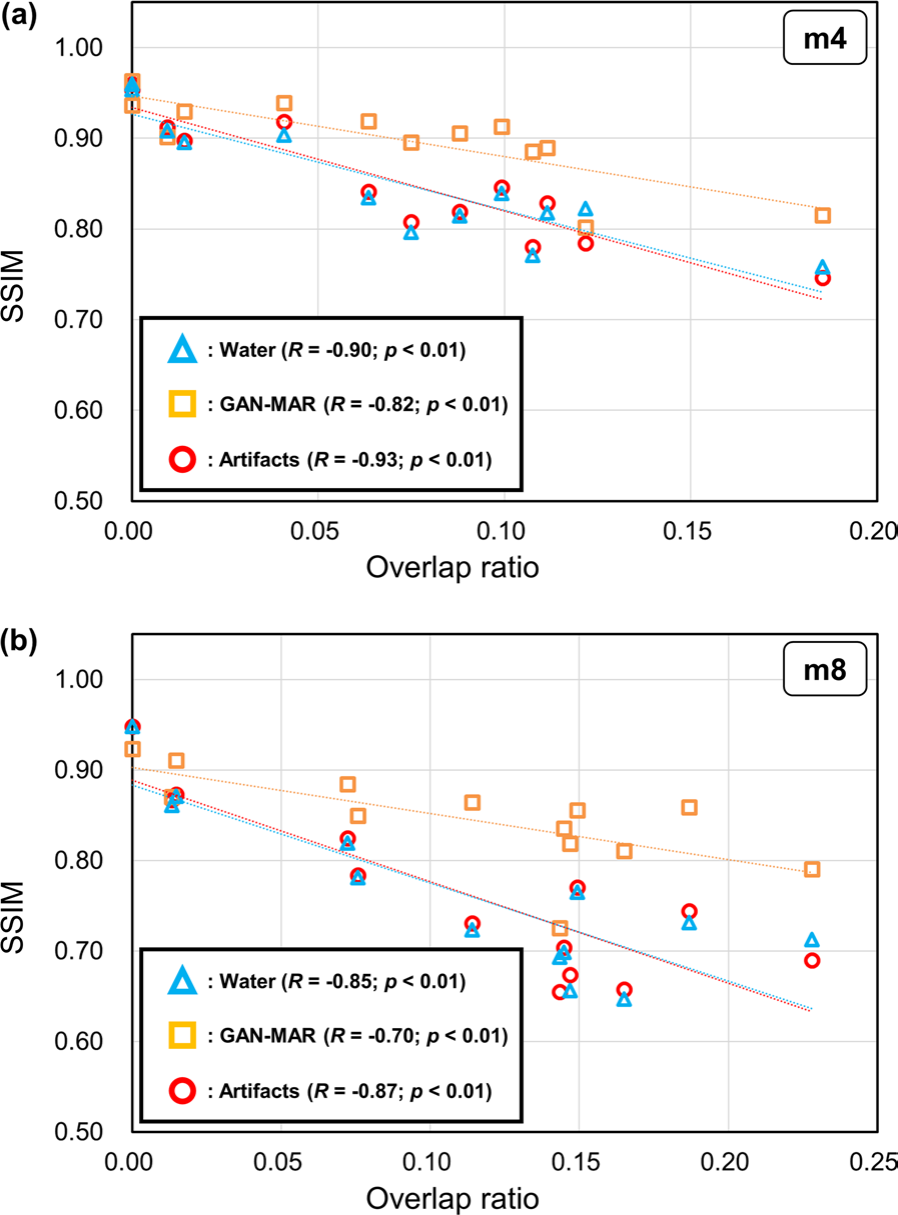
SSIM as a function of the overlap ratio of the artifact corrected volume to the PTV volume for the **a** m4 and **b** m8 groups

**Fig. 5:**
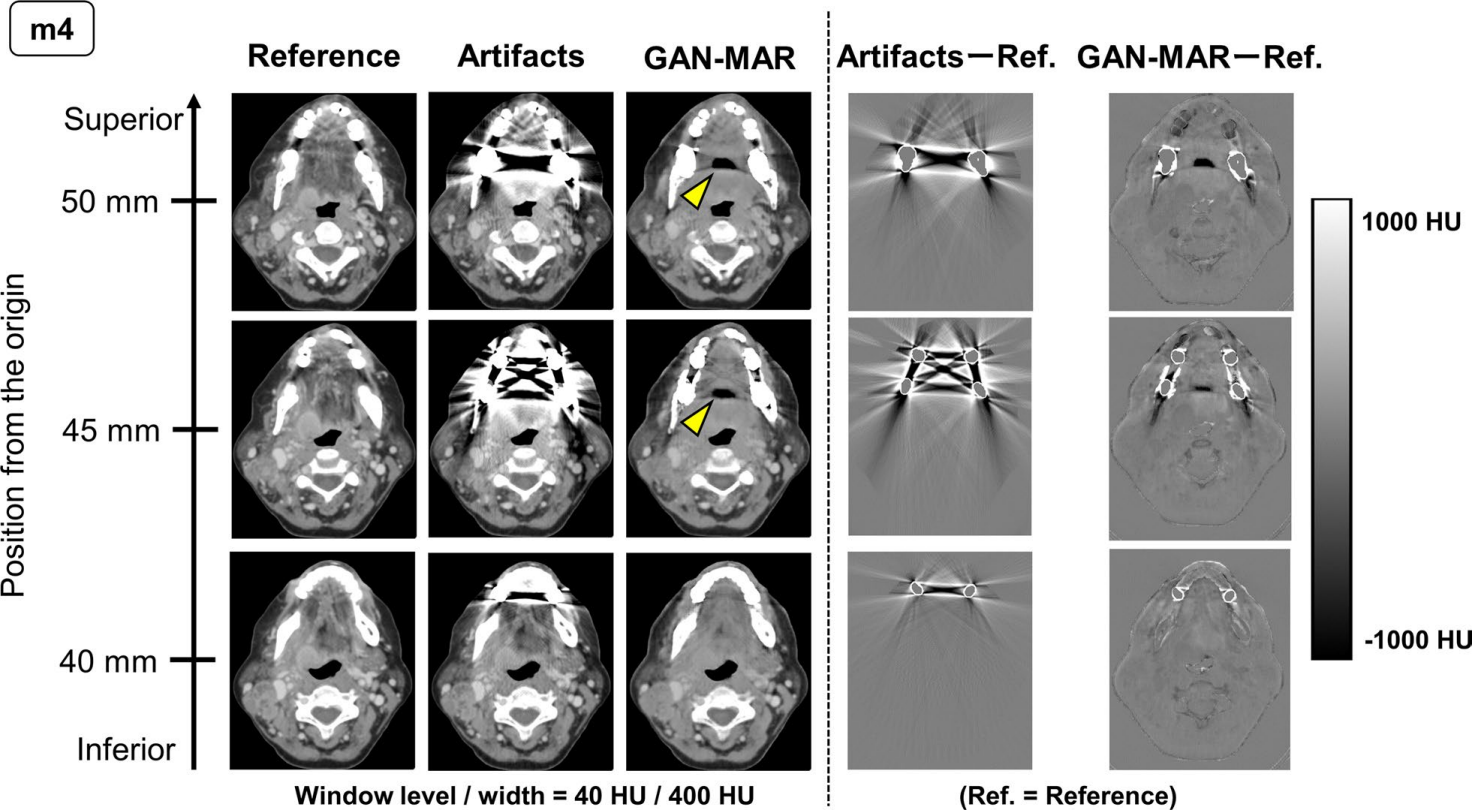
Axial slices for the case with the worst SSIM in GAN-MAR in the m4 group. The region indicated by the yellow arrow is the part of under-correction. Difference images to Reference are also shown

Figure 4 shows the SSIM as a function of the overlap ratio of the artifact corrected volume to the PTV volume. The SSIM exhibited negative correlation with the overlap ratio. The slopes for GAN-MAR were − 0.67 in the m4 group and − 0.51 for the m8 group. By contrast, slopes for Artifacts and Water were steeper than GAN-MAR, and Artifacts exhibited a similar tendency to Water. Among the thirteen cases, three in the m4 group and one in the m8 group exhibited lower SSIM than Water or Artifacts. One value in the m4 and m8 groups exhibited an overlap ratio of 0, which means that the water-corrected volumes were outside the PTV. At an overlap ratio of 0.122 in the m4 group, a drop in the SSIM in GAN-MAR was observed. Considering this case, under-correction parts (indicated by yellow arrows) were observed in the tongue, and metal artifacts were substantially reduced on the GAN-MAR CT volumes (Fig. 5). A drop in the SSIM in GAN-MAR was also observed at an overlap ratio of 0.143 in the m8 group, owing to the same reason. However, the SSIM was higher than that in Water and Artifacts (Fig. 4b).

Table 2 summarizes the difference in DVIs from the reference plan for the PTV. Although the range between the minimum and maximum values broadened in the m8 group compared to the m4 group, the differences in DVIs from the reference plan were within 3% in the VMAT plans and 1% in the IMPT plans. The Mann–Whitney U test showed significant differences in all DVIs in VMAT and D_98%_ in IMPT (*p* < 0.05). No correlations were observed between the differences in the DVI, RMSE, and SSIM in the VMAT and IMPT plans. The difference in the D_max_ for the spinal cord and the D_mean_ for the parotids was less than 0.2 Gy.

**Table 2:** The difference in DVIs from the reference plan for the PTV

	VMAT			IMPT		
	D_98%_ (%)	D_50%_ (%)	D_2%_ (%)	D_98%_ (%)	D_50%_ (%)	D_2%_ (%)
*m4*						
Water	0.2 (− 0.7 to 0.8)	0.1 (0.0–0.3)	0.5 (0.0–1.1)	0.0 (0.0–0.4)	0.0 (0.0–0.0)	0.0 (0.0–0.0)
GAN-MAR	− 0.1 (− 3.0 to 0.1)	− 0.1 (− 0.2 to 0.1)	0.0 (− 0.2 to 0.0)	0.0 (− 0.2 to 0.1)	0.0 (0.0–0.0)	0.0 (0.0–0.0)
*p* value	< 0.05	< 0.05	< 0.05	< 0.05	< 0.05	< 0.05
*m8*						
Water	0.4 (0.1–0.9)	0.1 (0.0–0.3)	0.6 (0.0–1.1)	0.0 (− 0.3 to 0.4)	0.0 (0.0–0.0)	0.0 (− 0.1 to 0.0)
GAN-MAR	− 0.4 (− 2.4 to 0.0)	− 0.2 (− 0.5 to 0.1)	− 0.1 (− 0.4 to 0.0)	− 0.3 (− 0.8 to 0.2)	0.0 (0.0–0.0)	0.0 (0.0–0.0)
*p* value	< 0.05	0.29	0.70	< 0.05	0.62	0.68

Data are shown in median (minimum–maximum)

*DVI* dose-volumetric index, *PTV* planning target volume, *VMAT* volumetric-modulated arc therapy, *IMPT* intensity-modulated proton therapy, *D*_*xx%*_ dose covering xx% of the volume

## Discussion

More than 90% of head and neck cancer are squamous cell carcinomas, and the growth rate of squamous cell carcinoma is faster than that of adenocarcinoma. Thus, it is preferable to treat head and neck cancer patients promptly. In clinical practice, some head and neck cancer patients undergo tooth extraction prior to radiotherapy to avoid severe metal artifacts, which affect quality of life and cause delays in starting radiotherapy. Therefore, reduction of metal artifacts is clinically essential in radiotherapy for head and neck cancer patients.

Considering the current clinical workflow, the Water CT volumes are typically used for anatomy segmentation and dose calculation. However, the Water CT volumes depend on how the metal artifacts are corrected by the observers, and they cannot be ground truths. We treated the artifact-free CT volumes with dental fillings as ground truths because it is thus possible to know the correct answer. When the differences in image quality and dosimetry are negligible from the reference, the results can be interpreted as the close to the reference.

Our GAN-MAR approach detects and reduces the partial volume where metal artifacts appear. This means that the corresponding partial volume was targeted for reduction. Although the regions with metal artifacts were generally well-corrected, a decrease in the image contrast was also observed on the other regions. In image reconstruction using GAN, the autoencoder, which is an algorithm to compress input data, retains only the important features and then restores the data to its original dimensions. In the translation process, the training principle makes autoencoders assign a high probability to training points; this cannot ensure that blurry points are assigned to a low probability. Accordingly, incorrect conversion of pixel values, appearing blurry to the human eye, would occur [15].

The lower SSIM in GAN-MAR than Water or Artifacts was due to the characteristics in the case with an overlap ratio of 0. One case exhibited a large deviation of the SSIM from the regression line in GAN-MAR in both the m4 and m8 groups (Fig. 4). As shown in Fig. 5, our approach failed to reduce the metal artifacts, as indicated by the yellow arrows. Because the dark band, which is indicated by the yellow arrow in Fig. 5, was similar to the feature of the air space in the oral cavity, the GAN generator *G* did not learn it as an image feature to be reduced but proceeded to learn it as a transformation that would preserve it. As a result, it passed the check of discriminator *D* because there was a similar air space in the real database. The under-correction could be improved by increasing such cases or the failure could be avoided if the MAR images are used for training our network and for input.

We have demonstrated that the difference in RMSEs and DVIs in the VMAT and IMPT plans was within 11 HU and 3%, respectively, for all situations considered in this study. There are several literatures that have conformed the validity of MAR algorithms for the head and neck region. Koike et al. reported the results of 2D evaluation using real patients’ CT volumes [17], and Branco et al. conducted a phantom study to assess the effect of clinically available MAR algorithms on 3D geometry and dosimetry [13]. Because our study was based on 3D analyses using artifact-free CT volumes of real patients with dental fillings as ground truths, direct comparisons could not be made with other studies due to different study settings, MAR methods and evaluation metrics. Nevertheless, the results of our study would be clinically acceptable when compared to those of previous studies [9–13, 17].

High *Z* materials produce dose backscatter, and dose reduction occurs downstream of the materials [22]. In addition to the dose disturbance, changes in the water equivalent path length greatly affects dose distribution in proton therapy [23]. These factors may yield inaccurate dose distribution at treatment planning, which leads to radiation-induced toxicities such as oral mucositis in head and neck cancer patients [3]. One possible reason for the small dosimetric difference can be the avoidance of incident photon and proton beams to the metal. The International Atomic Energy Agency (IAEA) quotes a requirement of 3% accuracy for calculated doses [24]. The IAEA [24] and the American Association of Physicists in Medicine (AAPM) [25] tolerance for accuracy of HU is 20 HU and 30 HU, respectively. Although evaluation metrics were different from the IAEA and AAPM, small dosimetric differences can be attributed to low RMSE of the HU values within the PTV. Interestingly, we also found that the water density override method commonly used is clinically acceptable.

Several limitations of this study warrant discussion. First, it is unknown whether our GAN-MAR is superior to the commercially available MAR algorithms; however, it is impossible to evaluate the effect of metal artifact correction and reduction in real patients with the commercially available solutions, due to lack of the artifact-free CT volumes with dental fillings. From the viewpoint of calculation time and image quality, our GAN-MAR would be equivalent or superior to the commercially available MAR algorithms. The second is the number of pseudo-dental fillings and HU values assigned. In this study, the number of pseudo-dental fillings was up to eight, and fixed HU values of 4000 HU were assigned to the pseudo-dental fillings. Among head and neck cancer patients, eight or more dental fillings were used, and their HU values may differ from 4000 HU, which may lead to the appearance of metal artifacts different from those in this study. The third limitation is the correction of metal artifacts. In general, manual correction procedures of metal artifacts are dependent on observers. In this study, one senior medical physicist conducted the correction, which may lead to a potential bias. However, we used two Artifacts CT images with different number of pseudo-dental fillings (four and eight) for each case and conducted geometric and dosimetric evaluation under the correction of metal artifacts. From the dosimetric results (Table 2), the effect of interobserver variability in artifact contorting on dose distribution can be negligible.

## Conclusions

This is the first study to evaluate the effect of metal artifact correction and reduction in terms of 3D geometry and dosimetry in radiotherapy for head and neck cancer patients, based on the artifact-free CT volumes with dental fillings. Our major findings can be summarized as follows: (1) The GAN-MAR CT volumes generated in a short time were closer to the Reference CT volumes than the Water and Artifacts CT volumes, and (2) the dosimetric difference in the PTV from the reference plan was within 3% in VMAT and IMPT.

## Data Availability

The datasets supporting the conclusions of this article are available from the corresponding author on reasonable request.

## Abbreviations

AAPM: American Association of Physicists in Medicine
ANOVA: Analysis of variance
CT: Computed tomography
CTV: Clinical target volume
CycleGAN: Cycle-consistent GAN
DL: Deep learning
DVI: Dose-volumetric index
GAN: Generative adversarial network
IAEA: International Atomic Energy Agency
IMPT: Intensity-modulated proton therapy
MAR: Metal artifact reduction
PTV: Planning target volume
RMSE: Root mean square error
ROI: Region of interest
SSIM: Structural similarity
VMAT: Volumetric-modulated arc therapy
2D: Two-dimensional
